# ChunkyBERT: a novel technique for multiclass political bias detection in news media

**DOI:** 10.1038/s41598-026-46646-z

**Published:** 2026-04-01

**Authors:** Daksh Loiya, Srinivas S. Kulal, M. Sai Mahith Reddy, Ramya D. Shetty

**Affiliations:** Manipal Institute of Technology, Manipal Academy of Higher Education, 576104 Manipal, India

**Keywords:** Political media bias, BERT model, Transformer encoding, Attention pooling mechanism, Multi-class classification, Information technology, Experimental evolution

## Abstract

With the increasing use of digital platforms for spread of information, political news has some of the most skewed sources which has confused people on what are facts and what are biased reportings. While most modern methods of finding bias in media use advanced Machine Learning algorithms and deep Learning techniques, such techniques heavily rely on manually generated features as sentiment analysis and lexical frequency which are tedious and time consuming. Hence we propose a novel method using Bidirectional Encoder Representations from Transformers (BERT) for ascertaining political media bias, mainly classifying articles into left-wing, centrist and right-wing inclinations via incorporation of the complete text. The suggested method includes dividing long political articles into segments of a set length. These are encoded separately using a pre-trained BERT model. A Transformer encoder then aggregates these segment-level embeddings, along with an attention pooling mechanism. This lets the model focus on and use the parts of the text that best show political bias for classification tasks. The experiments showed a highest validation accuracy of 86.22% and a validation AUC-ROC of 0.96, which is better than standard methods. This research gives a way to scale the detection of political bias, with potential uses in journalism, media monitoring, and improving digital literacy tools.

## Introduction

In the modern digital era, the large volume of data available about events, people and locations makes it impossible for an individual to identify biases in the media they consume. This is very true is media as they shape a large number of peoples sentiments and their democratic selection bias. The ways in which medias does this is by selection bias, where only certain details are provided. Framing, which modifies the interpretation of information via how it is presented to the viewer and finally by diction, where the emotionally manipulation is used for gaining sympathy and misleading the situation^1^. This massive capacity to skew public opinion is very important as it allows for ill-informed decision-making, and divides society as a whole. As people become more dependent on media outlets as news sources, the lack of ability to identify and understand underlying biases can spread misinformation, support already existing echo chambers, and further divide communities, which might cause communities to become fractured further, as well as result in the proliferation of misinformation and circulation of falsehoods^2^. In democratic societies, the consequences are horrifying, as a misinformed decision making body is an actual danger to the entire democracy in general. Media bias detection has been a promising aspect of conventional machine learning (ML) approaches to detecting this bias and more recently advanced deep learning (DL) models.

The classical machine learning techniques apply linguistic features, such as sentiment scores, lexical frequencies, and named entities, that are manually obtained and serve as inputs to the classifiers, such as support vector machines (SVMs)^3^. Although the techniques provide explainability, they rely on defined features, and they are unable to find the contextual information effectively. BERT methods of deep learning^4^ have proven to be effective. These models derive contextual representations, aiding the prediction of bias, of a raw text, based on semantic relationships. Convolutional neural networks (CNNs) and recurrent neural networks (RNNs) models have been demonstrated to be useful in sequential data analysis to identify language patterns that can be used to detect bias, as well as in bias detection^5^. The combination of the two approaches is the hybrid methods that integrate traditional machine learning properties with features of deep learning embeddings that can integrate the advantages of both approaches and provide more robust and complete solution to the problem of media bias.

Our new approach has a chunk-based BERT model to examine the news items based on their political biases, which can be left, center, or right. In it, a data preprocessing module, or head, is used that splits articles into smaller segments and tokenizes them. Such chunking method is crucial, because it enables the model to cope with longer articles, and aid in the subtle information about the context, usually involved in politics discourse. A multi-stage model design used the chunk-level BERT encoder contextual embeddings, followed by a Transformer encoder. An attention pooling layer measures the importance of each token, allowing the model to focus on words or phrases that are most indicative of the article’s political bias.

The rapid rise of digital media has changed news consumption behaviours, thus leading to a rise in problems based on political media bias. Biases like these distort public opinion, deepen political and ideological divides, and lower journalistic institution’s confidence^6–8^.. As digital platforms start to have a greater impact on public discourse, this requires additional research to examine automated, scalable, and accurate systems engineered to detect such biases.

### Our contributions

- We introduced a *Chunked BERT-based *design for the detection of political media bias, which processes long-form news articles through chunking, while maintaining contextual information.
- We experimented with a hierarchical aggregation strategy that uses Transformer encoder with an attention pooling mechanism to model dependencies between chunks and highlights textual segments that are the most important of ideological bias.
- We conduct experimental tests through multi-class data (left, center, right), to illustrate that our proposed approach results in state-of-the-art accuracy and AUC-ROC scores against the traditional machine learning methods and deep learning models.
- We offer scalable and generalizable framework to be used in practical media monitoring systems, journalistic analysis, and digital literacy initiatives.

The structure of the paper is the following: Section “Related works” entails in-depth analysis of recent researches on the field of media bias detection and natural language processing, focusing on the existing methodology, its benefits, and limitations. Our recommended methodology is described in Section “Methodology” with details of the model architecture, data distribution, training schemes, and experimental procedures. Section "Results and discussions" discusses the findings of our experiments, evaluates the usefulness of our method and how it can be applicable to the field of political media analysis. Lastly, Section “Conclusion” wraps up the paper with outlooks on the further work, such as the additions aimed towards an improved bias detection.

## Related works

### Hate speech and misinformation detection

Recent works have moved beyond the conventional linguistic characteristics to the contextualized deep learning methods of harmful content detection. Transformer embedding models coupled with hierarchies have been shown to be very effective at detecting explicit and implicit, context-specific hate speech instantiations in their most subtle forms, as well as in identifying novel and unanticipated forms of hate speech that may emerge in the future, including the use of new AI languages^9^. Detection of satire is particularly tricky since it is an issue that is based on sarcasm, exaggeration, and contextual cues, especially in the short form of text, like in a tweet or a headline, and has close connections to misinformation and fake news. Older approaches that rely on characteristics and language inconsistency were typically weak in generalization, with recent research demonstrating that transformer-based deep learning models with attention-based biLSTM models are more capable of implicitly capturing the satirical intent in a text passage^10^. According to recent literature, contextual and domain-sensitive representations have been recently indicated as significant in the detection of fake news, and financial misinformation has received relatively low attention despite its implication in the real world. Combined with linguistic context models (e.g., BERT) that consist of domain-specific feature manipulations on top of attention-based sequential architectures are much more effective in identifying misleading content with reliability and precision^11^.

###  Sentiment analysis and bias quantification

Media bias has a notable influence on societal views, especially in subjects like politics, religion, and gender equality. Chauhan et al.^12^ used sentiment analysis with VADER and RoBERTa to analyse emotional tones in Indian mainstream news, highlighting significant political party, religious, and gender biases. Similarly, Choudhary et al.^13^ studied political leaning in relation to Large Language Models (LLMs), revealing that commonly used commercial LLMs such as ChatGPT, Claude, and Perplexity exhibit a liberal bias. Tankard Jr. et al.^14^ suggested a bias detection framework using Twitter that uses sentiment analysis and Natural Language Processing (NLP) to determine a “bias score” based on negative sentiment tweets, notably those with offensive language and related to important people or concepts. Sharma et al.^15^ examined political bias in Indian TV news channels, analyzing their Twitter activity through sentiment analysis using Naïve Bayes and Majority Voting algorithms. Their results showed positive and negative biases in political parties. Hijazi et al.^16^ presented a lexicon-based sentiment analysis model for the Sabah dialect of Malay, enabling bias-aware thresholding and dialect-specific lexicon changes to attain high accuracy in language sentiment classification. Patankar^17^ created a real-time system for spotting biased language in news articles by assessing word vector similarity against a bias lexicon. This method allowed the system to give article bias scores, differentiating between objective reporting and opinionated content, and was made into a browser extension for user access.

### Machine learning and NLP for bias detection

Benson^18^ created an unsupervised machine learning approach that pinpoints media bias in cable news programs, employing Named Entity Recognition and Stance Analysis Or Bias and grouping programs with similar biases. This showed consistent bias patterns in particular networks. Fatima^19^ assessed BERT, logistic regression, and LSTM-based models to measure media bias across mainstream, conservative, and liberal sources, with BERT demonstrating the strongest performance. Swati^20^ applied IC-BAIT, a neural network framework, to spot ideological bias in news headlines, focusing only on textual data. Zahid et al.^21^ analysed media tweets utilising matrix-based data modelling and statistical metrics to assess coverage and statements. Spinde et al.^22^ introduced a dataset of 1,700 annotated statements and a feature-driven bias detection model, outperforming previous methods by an F1-score of 0.43 and AUC of 0.79. Rodrigo-Ginés^23^ conducted a review on media bias literature, cataloguing 17 bias classifications and comparing 63 automated detection systems, coming to the conclusion that this domain still requires more research. Rakhecha^24^ surveyed natural language processing (NLP) approaches for bias detection, using deep learning and machine learning methodologies. Spinde^25^, again, uses NLP with deep learning to classify bias using linguistic features, but noted their dependence on labelled datasets. Dey^26^ implemented a Bag-of-Words model for fake news detection using linguistic traits, while discussing challenges of multilingual data. Azizov et al.^27^ implemented a CatBoost model to classify political bias in articles and media sources, boosting performance using ensemble techniques and dataset balancing. Baly^28^ formulated a balanced dataset for political bias, employing adversarial adaptation and triplet loss to help with model generalizability. In a related study, Baly et al.^29^ studied bias and factuality detection by social media interaction and audience demographics. Sharma et al.^5^ proposed a ReNN-based framework for bias detection in Indian news, detecting pro-policy tendencies and biases. Ruan et al.^7^ devised a triadic mechanism to identify and counteract bias in headlines employing BERT and PMI, preserving semantic and grammatical correctness. Sharma et al.^30^ implemented an ensemble model using CNNs and GRUs to detect political bias, capturing local and long-range dependencies. Baraniak et al.^31^ examined news article resemblance to explore media bias analysis through clustering and classification, studying the importance of source bias. Finally, Zhang^32^ presented a polarisation-aware graph embedding strategy for social media knowledge graphs, enabling bias modulation to balance fairness and performance across political and neutral contexts.

### Algorithmic bias in recommendations

Chen et al.^33^ studied algorithmic biases and their influence on content recommendation systems on media platforms. Their research explain methods such as collaborative filtering, content-based filtering, and hybrid models use behavioural data to personalise user recommendations. The work also discusses algorithmic prejudice, privacy concerns. Ruan et al.^8^ utilise existing news recommendation algorithms concerning their preference to hide the spectrum of viewpoints users encounter, which reduces public perception and impacts social decision-making. Their research studied models like NPA, NAML, LSTUR, NRMS, FIM, and PLM on the MIND dataset, revealing that users were presented content that was progressively skewed towards the content they interact with. Patankar et al.^34^ studied a diversity-conscious recommendation strategy to tackle political bias by exposing users to an expanded set of viewpoints. While their framework has not implemented fake news detection, they plan on integrating publicly accessible news credibility or fake news identification APIs in the future to refine the computed bias scores.

### Large Language Models (LLMs) and multimodal approaches

Kuntur et al.^35^ explored the importance of dealing with misinformation,especially after the 2016 U.S. presidential election, where false stories influenced public opinion. While LLMs make fake news detection harder by making it possible to create very convincing and logical content, the authors suggest that these models also open up new defensive opportunities such as adaptive learning systems, multimodal integration, and near-real-time monitoring of misinformation.

Do et al.^36^ present GDMFN, a general structure for fake news detection that models both textual content and social context. Their method combines basic and advanced representation methods like word2vec, doc2vec, and transformer-based embeddings along with bias detection, clickbait detection, sentiment analysis, and toxicity classification. By using graph convolutional networks and mean-field layers to understand structural relationships, GDMFN performs better than current baselines on Twitter, Weibo, and PHEME datasets. Singhal et al.^37^ tackle the increase in multimodal misinformation by suggesting SpotFake, a unified model created to manage multimodal data. Their design uses BERT to pull out semantic textual features and VGG19 for image processing. Experimental results indicate that SpotFake achieves performance increases of 3.27% on Twitter data and 6.83% on Weibo. Lastly, Silva et al.^38^ introduce $\textrm{UMD}^2$, an unsupervised multimodal misinformation detection framework that works across textual, user, and temporal aspects. By not needing labeled data, they have observed a 6–8% accuracy improvement over GTUT graph-mining baseline, emphasizing the effectiveness and scalability of unsupervised strategies for misinformation detection.

### Literature summary

Studies into fake news detection and media bias analysis show that, despite the advantages of each method, solutions with great accuracy and precision are limited. Research into sentiment and bias analysis often does not consider the interaction between them, which can help reinforce current views. Supervised models need labelled datasets, which limits how scalable and adoptable these models can be, making it hard for them to adapt to the constantly changing world of misinformation. Multimodal deep learning methods show promise, but often have issues with high computational costs and problems handling multilingual content. Public trust can be undermined by the lack of transparency in how many models decide things. As a result, a better strategy would be to combine fake news detection with bias mitigation and use data sources.

**Table 1:** Comparison of recent approaches for fake news detection and media bias analysis as part of a survey of current literature in our research domain.

Method	Reference (Year)	Main Features	Limitations
Sentiment-based Bias Detection	Chauhan et al. (2022)^12^	Uses sentiment analysis with VADER and RoBERTa to quantify emotional bias in Indian mainstream news, revealing gender, religious, and political slants.	Relies heavily on sentiment polarity; limited in capturing deeper ideological framing and contextual bias.
Neural Bias Detection using Transformers	Spinde et al. (2021)^22^	Introduces a labeled dataset of political statements and applies neural NLP models for bias detection, achieving competitive F1 and AUC scores.	Performance depends on annotated datasets; struggles with scalability and cross-domain generalization.
Ensemble Deep Learning for Ideological Bias	Sharma et al. (2021)^30^	Proposes an ensemble of CNN and GRU models to capture both local textual cues and long-range dependencies for political bias classification.	Requires large labeled datasets and significant computational resources; limited explainability.
LLM Bias Analysis	Trhlík and Stenetorp (2024)^39^	Analyzes political bias in Large Language Model generated news articles using a curated corpus of real and synthetic news, enabling systematic comparison across LLMs.	Focuses mainly on English news and fixed topics; bias behavior may change with rapid LLM updates.
Multimodal Fake News Detection	Zhu et al. (2025)^40^	Introduces the MFND dataset and a shallow–deep multitask learning framework that jointly analyzes textual and visual cues to detect and localize fake news.	Primarily evaluated on curated multimodal datasets; generalization to real-world scenarios remains challenging.
Algorithmic Bias in Recommendations	Ye et al. (2025)^41^	Conducts a large-scale audit of social media recommendation systems using controlled accounts to quantify political exposure bias during elections.	Limited to short temporal windows and non-interactive accounts; personalization effects are not fully captured.
**ChunkyBERT (Proposed Method)**	**Our Work**	**Implements long-form political news bias classification using a hierarchical BERT-based architecture with fixed-length chunking and attention pooling**	**Primarily evaluated on U.S.-based news sources and reflects Western political bias definitions**

**Fig. 1:**
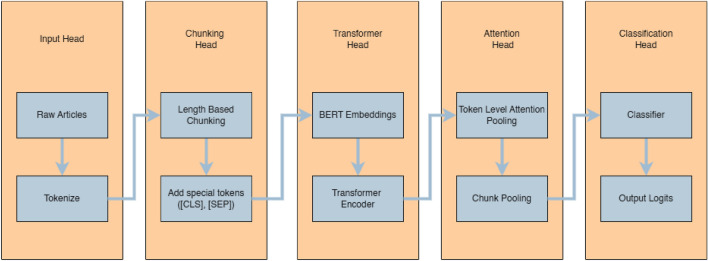
Workflow of the proposed ChunkyBERT framework for political media bias detection, consisting of five “heads” or modules: Input Head for article input, Chunking Head for article chunking, Transformer Head for BERT encoding, Attention Head for highlighting tokens using model attention weights and Classification Head for article classification.

## Methodology

### Overview

The objective of this research is to accurately classify long political news articles according to their media bias, namely *Left*, *Centre*, and *Right*. Political news articles are often lengthy and contain subtle ideological cues distributed across the entire document, making bias detection a challenging task. Transformer-based language models such as BERT impose a strict upper bound on input length, limiting their direct applicability to long-form text.

The overall workflow of the proposed approach is illustrated in Fig. 1. The methodology follows a multi-stage pipeline comprising data preprocessing, chunk-based encoding, hierarchical representation learning, attention-based aggregation, and document-level classification. The model is trained end-to-end and evaluated using standard classification metrics to ensure a robust assessment.

### Proposed model architecture

An overview of the model architecture is shown in Fig. 3, while the algorithmic flow is described in Algorithm 1.

#### Input encoding and chunking head

**Fig. 2:**
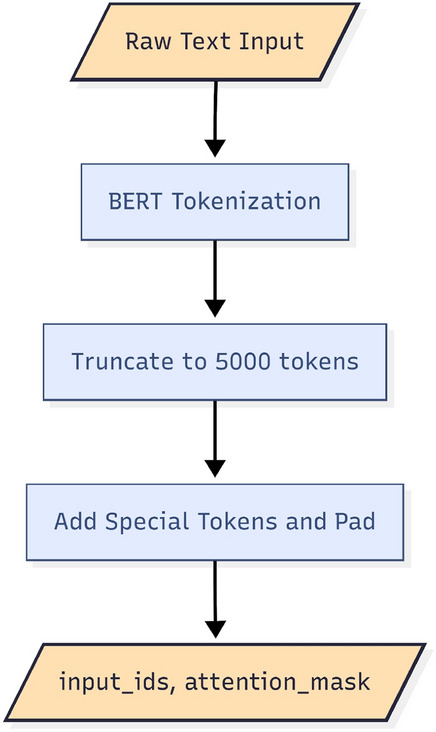
Tokenization and fixed-length truncation process applied to long political news articles when passed through the Input Head.

Each document is tokenised using a BERT tokeniser, as illustrated in Fig. 2. We have used truncation to the first 5000 tokens to balance computational time and information. Since BERT only allows a maximum input length of 512 tokens, fixed-length chunks are obtained based on token count. Sentence or paragraph boundaries are not considered, and no overlap is introduced between chunks. This design allows predictable memory usage and avoids heuristic segmentation strategies that might lead to unintended bias.

Given a document $X_i$, chunking gives:

$$X_i \rightarrow \{X_{i,1}, X_{i,2}, \dots , X_{i,K_i}\},$$

where each chunk contains at most $L = 512$ tokens, including special tokens.

#### Hierarchical transformer encoding head

Each chunk $X_{i,j}$ is encoded using a pretrained BERT model:

$$H_{i,j} = \mathcal {B}(X_{i,j}, M_{i,j}),$$

where $\mathcal {B}(\cdot )$ represents the BERT encoder and $H_{i,j} \in \mathbb {R}^{L \times d}$ represents token embeddings. The BERT outputs are passed through a Transformer encoder $\mathcal {T}_e$:

$$H'_{i,j} = \mathcal {T}_e(H_{i,j}),$$

which provides additional contextual refinement.

#### Token-level attention pooling head

Instead of relying on the [CLS] token, token-level attention pooling is applied to summarise each chunk. Attention scores are computed as:

$$\alpha _{i,j,t} = \text {softmax}_t \left( \textbf{w}^{\top } \sigma (\textbf{W} H'_{i,j,t}) \right) ,$$

where $\textbf{W}$ and $\textbf{w}$ are learnable parameters and $\sigma (\cdot )$ denotes the GELU activation function.

The chunk representation is obtained as:

$$Z_{i,j} = \sum _{t=1}^{L} \alpha _{i,j,t} H'_{i,j,t}.$$

**Fig. 3:**
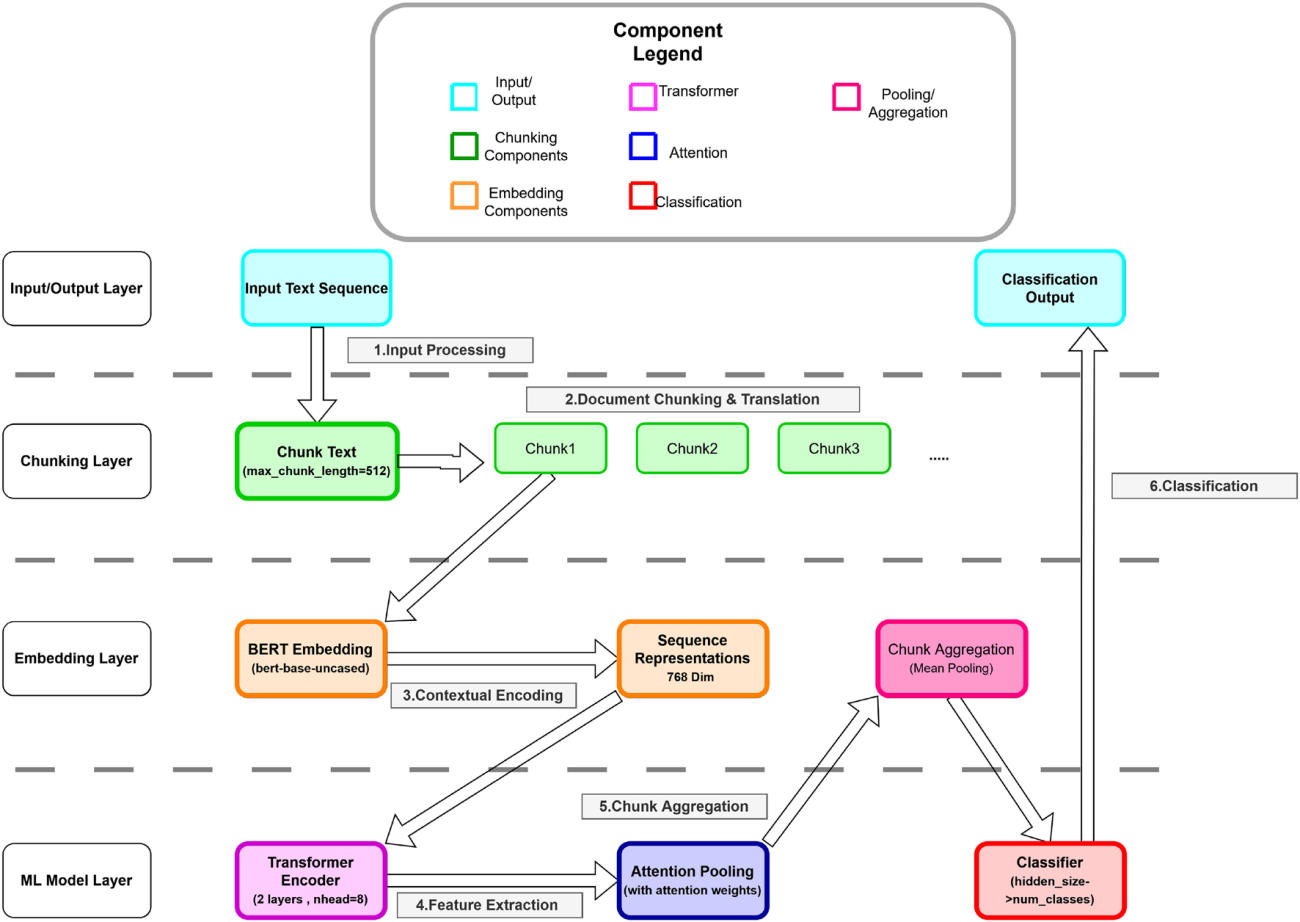
Architectural overview of the proposed ChunkyBERT model showcasing the inner mechanism of the model, divided into functional layers: Input/Output Layer for article entry and exit, Chunking Layer for chunking mechanism, Embedding Layer for obtaining embeddings and weights, and Model Layer for using said embeddings for bias calculation.

Unlike mean or max pooling, attention pooling enables the model to emphasize ideologically salient tokens while suppressing less informative content, which is particularly beneficial for long political texts (Fig. 3).

#### Document-level classification head

For each document $X_i$, chunk representations are aggregated using mean pooling:

$$Z_i = \frac{1}{K_i} \sum _{j=1}^{K_i} Z_{i,j}.$$

The resulting document-level embedding is passed through a classification head consisting of dropout followed by a linear layer:

$$Y_i = \mathcal {C}(Z_i),$$

where $Y_i \in \mathbb {R}^{C}$ represents the logits for the $C$ bias classes.

**Algorithm 1:**
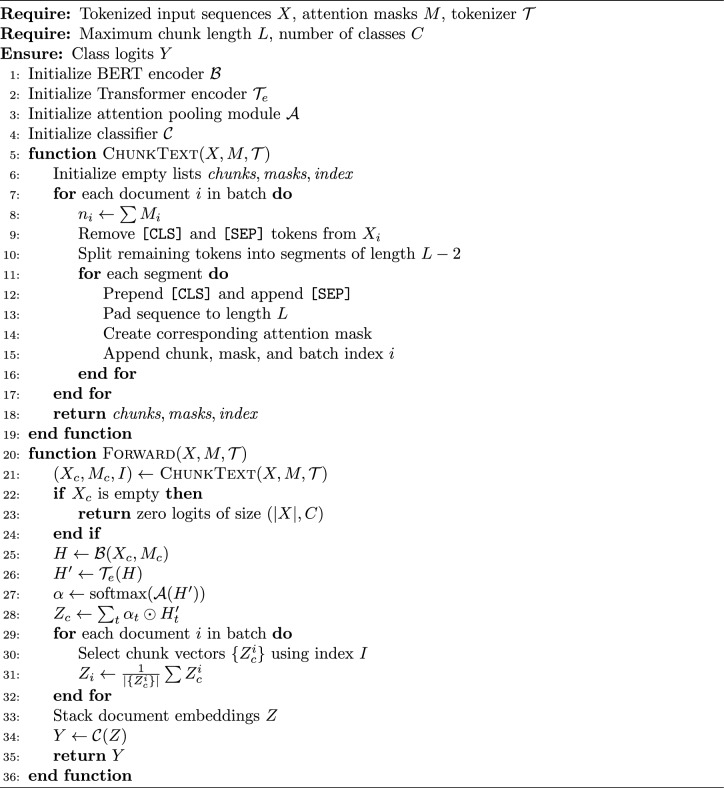
ChunkyBERT Classifier for Long-Document Multiclass Political Bias Classification

### Dataset properties and class distribution

The dataset^42^ consists of 37,554 news articles, each described by seven columns. It includes metadata such as the article’s link, title, source, and author, along with the full content and a numeric label indicating the article’s political bias. The dataset is well-structured and mostly complete, with only one column author having missing values (approximately 25.7% missing). The most informative columns for tasks such as text classification or media bias detection are likely to be title, content, and bias. Meanwhile, source may help identify patterns of bias across different news outlets. Overall, the dataset is clean and well-suited for natural language processing tasks related to fake news, media bias analysis, or source credibility assessment. All the above statisitcs are mentioned in the Table 2

**Fig. 4:**
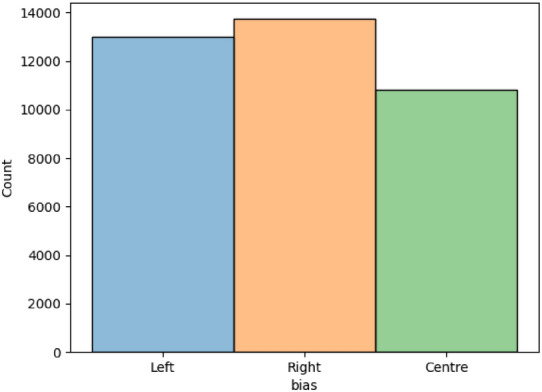
Histogram showcasing uniform distribution of political bias classes (Left, centre, Right) in the dataset.

The dataset exhibits a near-uniform class distribution, as seen in Fig. 4, with an imbalance ratio of 1.27 between the largest and smallest classes. Given this mild imbalance, no explicit class rebalancing techniques are applied.

**Table 2:** Summary of metadata attributes and missing value statistics of the dataset used for our experiment.

Column Name	Non-Null Count	Datatype	Missing Values	Missing (%)
link	37554	object	0	0.00
title	37554	object	0	0.00
content	37554	object	0	0.00
bias	37554	int64	0	0.00
source	37554	object	0	0.00
author	27886	object	9668	25.74

**Table 3:** Class-wise sample distribution of political bias labels of the dataset used.

Class	Samples
0 (Left/Progressive Leaning)	13005
1 (Centre/Moderate Leaning)	10815
2 (Right/Conservative Leaning)	13734

### Training strategy

The model is trained end-to-end using categorical cross-entropy loss, which is suitable for multi-class classification tasks:

$$\mathcal {L} = - \sum _{c=1}^{C} y_c \log (\hat{y}_c),$$

where $y_c$ is the ground-truth label and $\hat{y}_c$ is the predicted probability for class $c$.

Optimization is performed using the AdamW optimizer with weight decay to improve generalization. A linear learning rate scheduler with warm-up steps is employed to ensure stable convergence. Dropout is applied within the Transformer encoder and classification head to mitigate overfitting. Early stopping based on validation performance is used to prevent unnecessary training once convergence is achieved. Mixed-precision training is adopted to reduce GPU memory usage and accelerate training. To counteract overfitting, we have employed early stopping mechanisms, which terminate training once the validation loss of the model starts to stagnate.

### Experimental setup

Documents exceeding BERT’s input limit are segmented into fixed-length chunks of 512 tokens. Shorter chunks are padded to maintain uniform input dimensions across batches. Training is conducted using a batch size of 16 for 5 epochs, with a learning rate of $2 \times 10^{-5}$ and a dropout rate of 0.3, which were empirically chosen based on validation performance to ensure stable convergence and effective regularization. The dataset has been divided into two splits, one for training, which uses 80% of the data, around 30,000 samples, and the rest of the data (7554 samples) are used for validation.

**Table 4:** Hyperparameter settings and configurations used during training of ChunkyBERT model.

Hyperparameter	Value
Pretrained Encoder	bert-base-uncased
Maximum Chunk Length	512 tokens
Maximum Document Length	$\sim$5,000 tokens
Batch Size	16
Learning Rate	$2 \times 10^{-5}$
Dropout Rate	0.3
Optimizer	AdamW
Training Epochs	5
Transformer Layers	2
Attention Hidden Size	256
Training Samples	30000 (80% of data)
Validation Samples	7554 (20% of data)

All experiments were implemented using PyTorch and the HuggingFace Transformers library. Training and evaluation were performed on the Kaggle platform utilizing NVIDIA Tesla T4 GPUs with 16 GB memory. Automatic mixed-precision (AMP) training was enabled to improve computational efficiency.

### Evaluation metrics

Table 5 presents the metrics used in this study along with their mathematical formulations where True Positive (TP) corresponds to instances correctly assigned to a given class, while a True Negative (TN) refers to instances correctly identified as not belonging to that class. A False Positive (FP) occurs when an instance is incorrectly assigned to a class, whereas a False Negative (FN) denotes instances that belong to a class but are incorrectly predicted as another class.

**Table 5:** Evaluation metrics used during model validation and their corresponding mathematical definitions.

Metric	Formula
Accuracy	$\displaystyle \frac{TP + TN}{TP + TN + FP + FN}$
Precision	$\displaystyle \frac{TP}{TP + FP}$
Recall	$\displaystyle \frac{TP}{TP + FN}$
F1-Score	$\displaystyle 2 \times \frac{\text {Precision} \times \text {Recall}}{\text {Precision} + \text {Recall}}$
AUC–ROC	Area under the ROC curve (TPR vs. FPR)

Accuracy measures the overall proportion of correctly classified instances. Precision and recall capture complementary aspects of classification performance by quantifying prediction correctness and coverage of positive samples, respectively. The F1-score provides a balanced measure by harmonically combining precision and recall. AUC–ROC evaluates the model’s ability to distinguish between classes and is independent of a fixed classification boundary. Macro-averaged metrics are reported to ensure equal contribution from all classes.

## Results and discussions

This section presents the detailed experimental results evaluated using various metrics. The training process spanned five epochs, with consistent improvements in both AUC-ROC and accuracy across epochs.

**Fig. 5:**
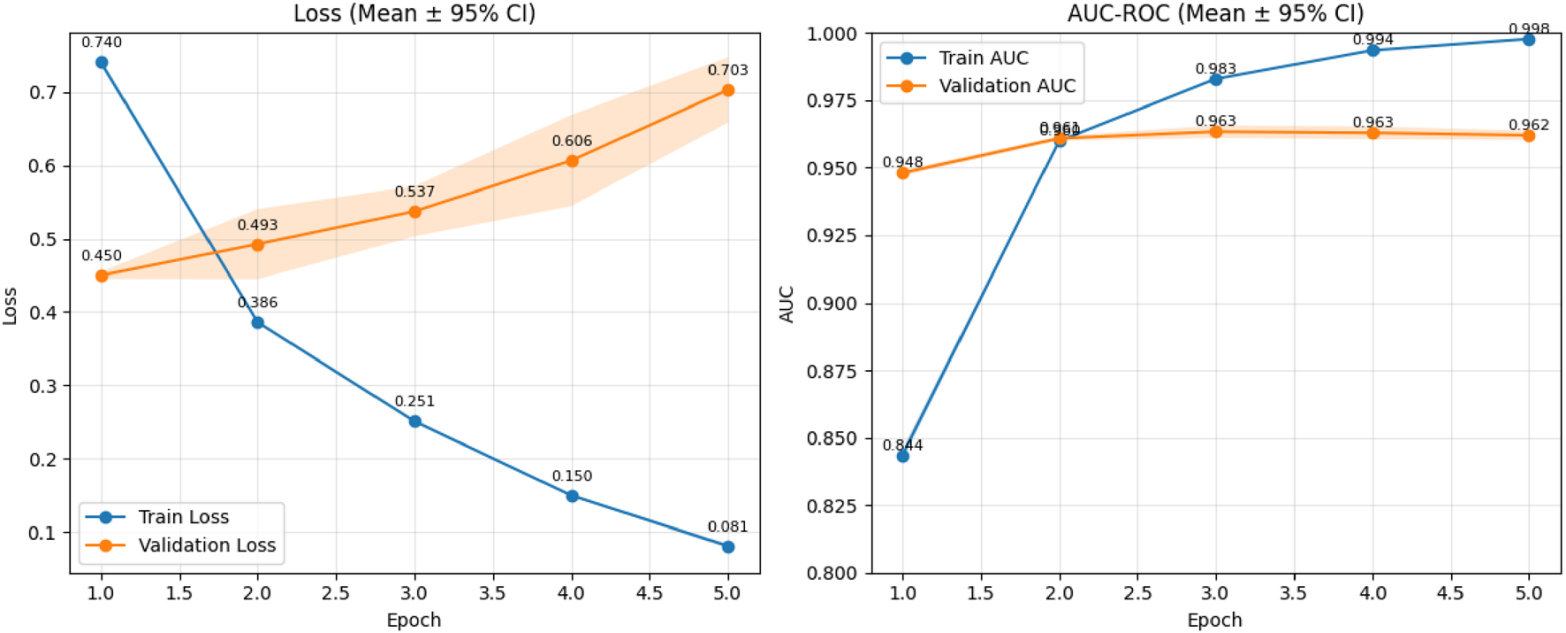
AUC–ROC and Loss curves of the ChunkyBERT model during training across five epochs showcasing best validation AUC-ROC of 0.962 at epoch 5 and visually representing the model overfitting after epoch 5, triggering the Early Stopping mechanism.

**Table 6:** Training and validation performance of ChunkyBERT across five epochs using training and validation model loss, AUC-ROC and Accuracy, showcasing consistent performance with Validation AUC-ROC of 0.962 and Accuracy of 86.22%.

Epoch	Train Loss	Val Loss	Train AUC-ROC	Val AUC-ROC	Val Accuracy (%)	CI, Std
1	0.740	0.450	0.844	0.948	83.91	[81.37, 84.74], 0.0128
2	0.386	0.493	0.963	0.960	84.01	[82.37, 85.71], 0.0237
3	0.251	0.537	0.983	0.963	85.02	[83.06, 85.68], 0.0116
4	0.150	0.606	0.994	0.963	85.30	[84.66, 86.85], 0.0052
5	0.081	0.703	0.998	0.962	86.22	[85.48, 86.57], 0.0048

### Model performance

As seen in Fig. 5 and Table 6, the model showed a quick and significant improvement in training loss and AUC-ROC during the first few epochs. The primary training and validation measures, such as loss, AUC-ROC scores, and validation accuracy, are shown in Table 6. Training AUC-ROC crosses 0.99 by epoch 4, showcasing the model’s ability to differentiate between the ideological classes well. Validation AUC-ROC shows strong performance, reaching a peak AUC-ROC of 0.9646 in epoch 3. This information is also shown in Fig. 5.

Validation accuracy improved gradually, reaching a maximum of 86.22% at epoch 5. While validation loss began to increase slightly from epoch 3 onward, this divergence from the training loss suggests the onset of overfitting. However, the stability of AUC-ROC and validation accuracy during this phase indicates that the model maintained effective discrimination capabilities.

### Class-wise evaluation

**Table 7:** Class-wise precision, recall, and F1-score obtained by ChunkyBERT in the fifth epoch highlighting robustness of ChunkyBERT across all classes.

Class	Precision	Recall	F1 Score
0	0.82	0.91	0.864
1	0.85	0.88	0.863
2	0.92	0.81	0.860

**Fig. 6:**
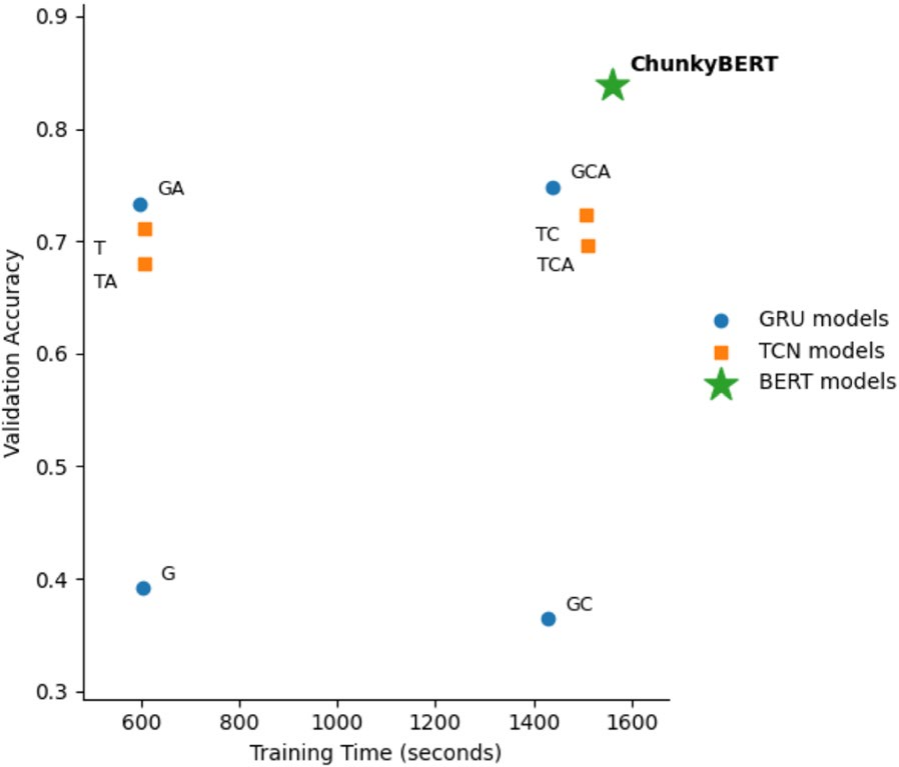
Training time versus validation accuracy for GRU-, TCN-, and BERT-based models, using base models, and using attention and/or chunking mechanism. ChunkyBERT outperforming other models without significant training-time. (G: GRU, T: TCN, C: Chunking, A: Attention, ChunkyBERT: ChunkyBERT).

To understand bias-specific performance, precision, recall, and F1-scores were computed for each class (Left, centre, Right). The results revealed the following trends and its shown in the Table. 7.

- **Left**: High precision and recall, suggesting the model accurately identified left-leaning rhetoric, likely due to consistent linguistic and framing patterns in the data.
- **Centre**: Slightly lower recall, indicating occasional misclassification as Left or Right, potentially due to the moderate and less distinctive tone of centrist articles.
- **Right**: High recall but slightly lower precision, possibly because certain centrist or nationalistic content was occasionally interpreted as right-leaning.

A normalized classification report (please refer to Table 7) was generated to visualize classification tendencies and misclassifications hotspots. Most predictions aligned with true labels, but some confusion was observed between the centre and the extremes. This supports the hypothesis that centrist articles often share overlapping features with both sides, complicating their classification.

### Training stability and generalization

Despite slight overfitting in the final epochs as seen in the widening gap between train and validation loss as shown in Table 6 and Fig. 5, the model’s validation performance remained robust. Early stopping based on AUC-ROC effectively mitigated excessive overfitting. Mixed-precision training significantly reduced training time and GPU memory usage without sacrificing performance.

### Comparision with different model architectures

**Table 8:** Checklist comparison of model architectures with respect to chunking, attention, training time, and accuracy obtained when training for one epoch, with ChunkyBERT outperforming all other model configurations without trading off in training time.

Model	Chunking	Attention	Architecture	Time (s)	Accuracy (val, %)
GRU no chunk, no attn	✗	✗	GRU	602.81	39.16
GRU no chunk, attn	✗	✓	GRU + Attn	596.94	73.29
GRU with chunk, no attn	✓	✗	GRU	1429.21	36.44
GRU with chunk, attn	✓	✓	GRU + Attn	1438.28	74.82
TCN no chunk, no attn	✗	✗	TCN	606.79	71.11
TCN no chunk, attn	✗	✓	TCN + Attn	607.46	68.02
TCN with chunk, no attn	✓	✗	TCN	1508.35	72.28
TCN with chunk, attn	✓	✓	TCN + Attn	1511.21	69.62
**ChunkyBERT**	✓	✓	BERT + Transformer + Attn	**1560**	**83.91**

**Table 9:** Comparison of ChunkyBERT with existing political bias detection approaches on using the same dataset.

Feature	Azizov et al. (2023) (Frank at CheckThat)	Baly et al. (2020) (What Was Written vs. Who Read It)	Baly et al. (2020) (We Can Detect Your Bias)	ChunkyBERT
Task	Detecting political bias in news articles and media outlets	Predicting political bias and factuality of reporting of news media	Predicting the leading political ideology or bias of news articles	Detecting political bias in news articles and media outlets
Dataset Size	55,000 articles	864 news media outlets	34,737 articles	37,554 articles
Dataset Source	AllSides	Media Bias/Fact Check (MBFC)	^42^	^42^
Model	CatBoost, Majority Voting	BERT, SVM, Various ensembles combining different information sources	Adversarial Media Adaptation, Triplet Loss, Transformers	Chunked Input, BERT Encoder, Transformer Aggregation, Attention Pooling, Text Classification
Key Focus	Handling class imbalance, using majority voting to improve media-level classification	Modeling what media write, who reads it, and what’s written about media; combining linguistic content with social context	Preventing the model from learning to detect the source instead of political ideology, using media-level representation to improve article-level prediction	Detecting political bias solely from the content of the article and without incorporating media source and authors into account
Article-level Accuracy	69.4	62.27 (BERT Probabilities)	36.75 (BERT Article Baseline)	86.22

Table 8 presents a comprehensive comparison of various model architectures based on their structural components, including chunking strategies, attention mechanisms, and transformer integration. Additionally, we analyze the training times and classification accuracies by introducing structural modifications to the GRU^43^ and TCN^44^ models. GRUs are employed for their proven ability to model temporal dependencies in sequential data while being computationally more efficient than LSTMs. According to Qin et al., GRUs function as a “lightweight framework for sequence labeling” with effective kernel operations that preserve high performance^43^. In contrast, TCNs analyze complete sequences in parallel while preserving temporal ordering by utilizing dilated causal convolutions. According to Bai et al., ”a simple convolutional architecture outperforms canonical recurrent networks such as LSTMs across a diverse range of tasks and datasets”^44^, which makes TCNs an appealing substitute for tasks involving sequence modeling, such as text categorization.

In particular, we investigate various attention and chunking combinations merged with GRU and TCN architectures. The findings demonstrate that both GRU and TCN models perform noticeably better when attention is included. For example, accuracy increases from 0.3916 to 0.7329 when attention is added to a GRU without chunking. Similar to this, chunking frequently results in better performance, particularly when combined with attention, but it also tends to significantly lengthen training times. The accuracy of the GRU with chunking and attention is 0.7482, demonstrating the advantage of integrating both approaches. With an accuracy of 0.7228, the model with chunking and no attention outperforms other TCN-based variations, indicating that chunking might be more advantageous to TCNs than attention alone. The usefulness of our models is demonstrated by the suggested *ChunkyBERT* model, which combines chunking, attention mechanisms, and transformer layers, outperforming all other configurations and obtaining the maximum accuracy of 0.8391 without compromising training time. Additionally, a theoretical comparison between our suggested strategy and the other baseline approaches is given in Table 9. Additionally, Fig. 6 shows a comparison between training time and validation accuracy in various model setups.

### Ablation study

We have conducted an ablation study to measure the contribution of individual “heads” of *ChunkyBERT*. Each “head” has been removed or simplified, including transformer-based contextual encoding, token-level attention pooling, document chunking, chunk aggregation, encoder fine-tuning, and classifier depth. All variants are trained for one epoch under the same experimental setting. We have tabulated our results in Table 10.

The full *ChunkyBERT* model achieves the highest validation accuracy (84%) and serves as the reference. Removing token-level attention pooling (NoAttn) and simplifying the classification head (NoClass) lead to modest performance drops of 2–3%, indicating that while helpful, these components are secondary to contextual encoding. Replacing mean chunk aggregation with max pooling (MaxPool) has similar drops, suggesting that aggregating information across all chunks is preferable to focusing on a few important segments from each chunk.

Removing document chunking (NoChunk) reduces accuracy by 5%, proving that more contextual information increases model accuracy, instead of just focussing on the first 512 tokens. Freezing the BERT encoder (FrozenBERT) causes a major drop to 56%, showing that fine-tuning for bias classification is important. The most severe drop in validation accuracy is observed when all transformer layers are removed (NoTransformers), with accuracy falling to 41%, confirming that transformer-based contextual representations are the dominant contributor to performance.

While simpler variants reduce training and inference time (time taken to process a document during validation), they bring along significant accuracy losses. Overall, *ChunkyBERT* provides the best trade-off between effectiveness and efficiency, justifying the inclusion of all architectural components.

**Table 10:** Ablation study results showing the impact of architectural components on performance after training for one epoch. This shows that each head is providing a performance boost as compared to not using it, with the Transformer Head and Chunking Head being the most significant in boosting performance.

Model	Accuracy (val, %)	CI [Lower, Upper]	Std	Training Time (mins)
**Full Model**	**84**	**[81.37, 84.74]**	**0.0128**	**27**
No Attention	82	[80.72, 82.49]	0.0036	27
No Classifier	82	[80.32, 83.37]	0.0061	28
Max Pool	81	[79.18, 83.78]	0.0092	28
No Chunking	79	[78.48, 80.66]	0.0044	12
Freeze BERT	58	[57.42, 58.89]	0.0030	13
No Transformers	41	[39.97, 41.40]	0.0029	5

### Lexicographical analysis of misclassified articles

**Fig. 7:**
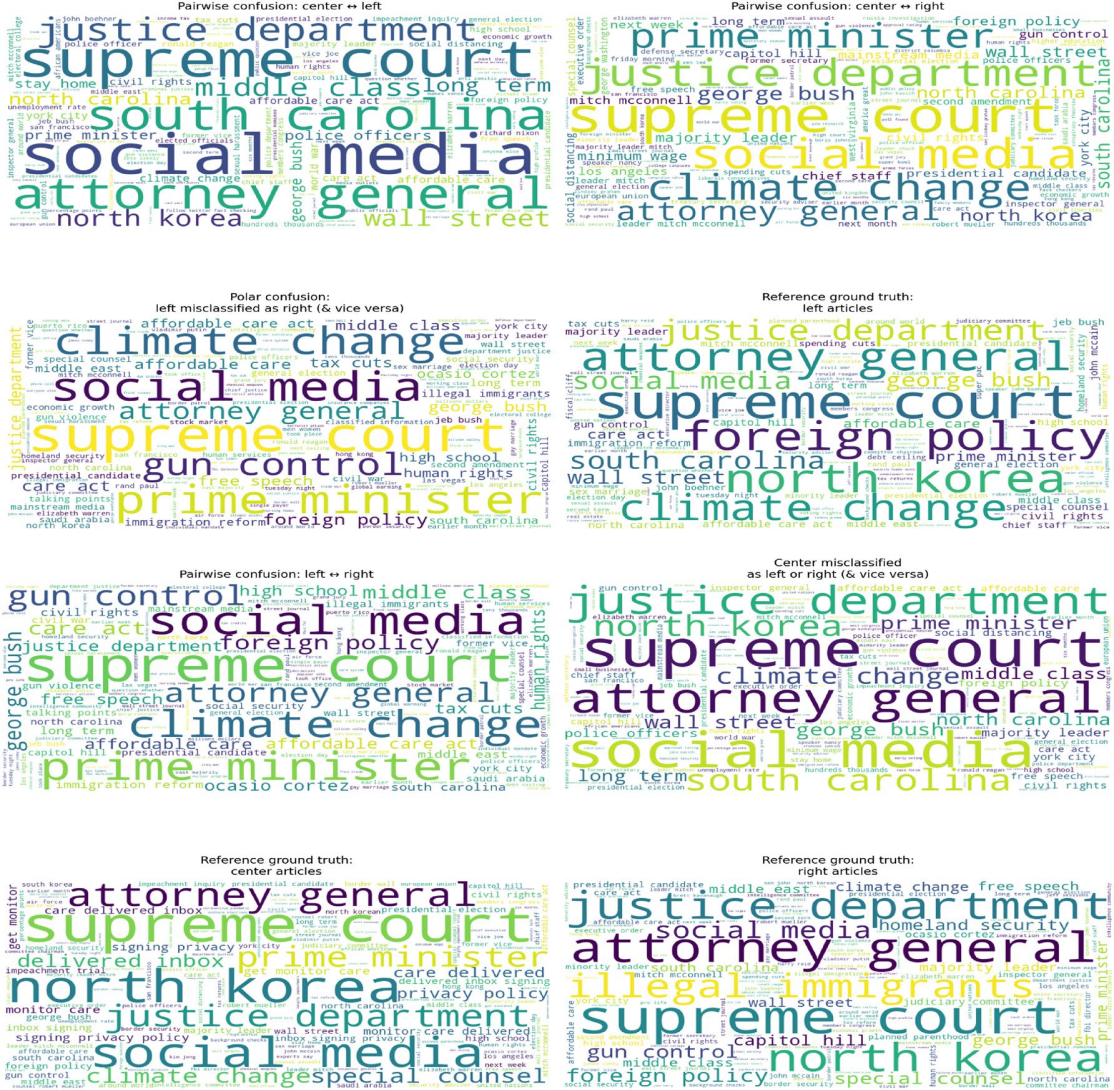
TF–IDF word clouds of bigrams and trigrams illustrating lexical patterns across political bias classes and misclassification types. The visualizations highlight substantial vocabulary overlap between classes and show that most classification errors occur between centrist and ideologically extreme articles, suggesting gradual lexical transitions across the bias spectrum.

TF–IDF weighted word clouds of bigrams and trigrams were constructed to examine the lexical characteristics of ground-truth bias classes and misclassified subsets after removing common stopwords and prominent political names. The class-level reference clouds reveal substantial lexical overlap across ideological categories, with recurring terms such as North Korea, Supreme Court, and Attorney General appearing across all classes. This indicates that political news discourse shares a broad topical vocabulary irrespective of ideological label, suggesting that class distinctions are driven less by exclusive keywords and more by contextual framing. Nonetheless, certain thematic patterns remain relatively class-associated, such as climate change and foreign policy in left-leaning articles, social media and privacy-related terms in centre articles, and topics such as illegal immigrants, gun control, and homeland security in right-leaning coverage. Together, these patterns imply that ideological differentiation emerges from nuanced emphasis and framing rather than discrete topical separation, as seen in Fig. 7.

Analysis of pair-wise and polar confusion clouds provides further insight into model behavior. Misclassifications involving centre articles (centre-left and centre-right) exhibit dense lexical blending, supporting the interpretation that centrist content occupies an intermediate semantic space where articles with weaker or mixed ideological framing are prone to boundary drift. In contrast, polar left-right confusions, while less frequent, contain strongly issue-driven vocabulary, indicating that ideologically opposed articles may converge lexically when discussing highly contentious topics. The dedicated centre-misclassification cloud additionally suggests that the model may over-rely on topical salience rather than framing cues, causing politically charged but neutrally framed articles to be pulled toward partisan classes. Overall, these observations indicate that the primary limitation lies in the feature representation’s ability to capture subtle framing differences near ideological boundaries,

### Limitations

*ChunkyBERT*, while effective in capturing nuanced language patterns, presents some limitations.

- The model’s application to political contexts in other areas of the world, particularly in Asia and Africa, is limited because it was trained on data primarily from American sources and will be skewed toward the political landscape, policies, and sociopolitical culture of that region. Its monolingual design also limits its use to English-language content, which makes it less useful in multilingual or global contexts.
- The majority of misclassifications occur in articles with weak, mixed, or subtle ideological cues especially close to the boundaries between the *centre* and *left/right* classes where neutral framing, balanced viewpoints, or selective policy emphasis obscure bias signals and cause unclear attention distributions across chunks.
- The discriminative power of content-based features is limited by linguistic neutrality in articles with high lexical and stylistic overlap across political categories. This suggests that residual misclassifications are primarily caused by inherent ambiguity in political discourse rather than model instability.

## Conclusion

This work presented ChunkyBERT, a chunk-based transformer architecture to classify political media bias, which is more predictive and interpretable. With contextual representation of transformers and chunk-scale aggregation and attention models, the model is able to capture both local and global linguistic and ideological structures in long-form political texts. The experimental outcomes indicate that ChunkyBERT outperforms classical machine learning methods and traditional neural models in all measures of evaluation, with a validation accuracy of 86.2% and validation AUC-ROC of 0.96. This work contributes to the development of political bias detection through the introduction of an empirically validated and robust framework that can process complex political discourse. The results demonstrate the usefulness of document modelling and language representations in contexts to reduce ambiguity and enhance credibility to media bias analysis, and highlights the importance of using the entirety of the article using chunking for boosting performance by ensuring no contextual meaning is lost.

Further, we have identified the following research areas for further domain exploration. Firstly, *Token-Level and Phrase-Level Attention Visualization* by using the attention weights obtained using the attention pooling phase, we have experimented with the model visualizing the weights for model explainability and to identify key tokens and phrases being used consistently by the model for bias prediction. With the growing importance of explainable AI, including this and attention visualization can lead to new ways to peek into the black-box, and warrants dedicated and further exploration. Secondly, *Domain Generalization and Multilingual Support* by multilingual and cross-cultural generalization is necessary, especially to regions that are underrepresented, as the data for non-English and non-Western media is limited and these populations in these areas are often targeted by biased media. Model compression methods will be considered to be efficiency-oriented to enable them to be deployed in real-time.

## Supplementary Information

Supplementary Information.

## Data Availability

The datasets generated during and/or analyzed during the current study are made available in the github repository, https://github.com/ramybaly/Article-Bias-Prediction. The model source-code is available in the github repository, https://github.com/dloiya/chunkybert.
